# A Word to Would-Be Students on the Course of Study

**Published:** 1900-09-08

**Authors:** 


### A Word to Would-be Students on the Course of Study.

Medicine is so vast a subject and touches so many sciences that the student who should attempt to browse undirected on the enormous field of knowledge laid before him would find that a lifetime instead of the allotted five years, would pass by before he attained an adequate knowledge of his profession. Thus, it has been found necessary to lay down a course of study which all must follow; and, although to some young spirits it may seem tedious and depressing that they should all have to tread in each other's footsteps through an unvarying curriculum, such a course is necessary, not only for their own sakes, but for the protection of the public.

The very first thing that the would-be medical student has to decide is the sort of qualification to which he aspires, a University degree or a diploma. This is a matter which is far too often overlooked. People imagine, and even schoolmasters, who ought, one would think, to know all about examinations and such like matters, seem to think that when a young fellow has got on a little way in his studies it will be time enough to decide in which direction his inclinations take him, and that it is sufficient for the present to pass the Preliminary and become enrolled on the books of a medical school. But this is not so, and one's choice of a Preliminary Examination all depends upon whether one seeks a diploma or a degree. If a University degree is desired one has but to follow the exact course laid down by the University which is selected, each University acting in this respect for itself. If, however, a diploma, say that given by the Conjoint Examining Board, be aimed at, what the student has to do is to pass one or other of the recognised Preliminary Examinations in Arts, a complete list of which can be obtained from the Registrar of the General Medical Council (299, Oxford Street, W.), or from the Registrar of the Branch Council in Scotland (48, George Square, Edinburgh), or Ireland (35, Dawson Street, Dublin). If he is wise, and it may be added if he is sufficiently well up, he will choose for this purpose the examination for matriculation at the University of London, since by so doing he will be able, should he afterwards lean towards a degree, to proceed to one without having to go back again to his preliminary studies. As soon as the Preliminary Examination has been passed the student should enter his name at the medical school to which he is going, and when this has been done he should without delay register at the office of the General Medical Council (299, Oxford Street, W.), as until this is done the five years of study which he has to undergo before he can enter for his final examination do not begin to count.

The student's career after registration may be roughly divided into three stages or periods. The first of these is devoted to elementary science, and during this period the student generally dissects; the second to anatomy and physiology; and the third to the purely professional subjects of medicine, surgery, midwifery, and the various specialties and departments into which they have become divided.

Let us consider the bearing on the student's life of this division into periods. The First Examination, which the student is expected to pass at the end of his first year of study, includes chemistry and physics, elementary biology, and practical pharmacy. It may be passed before registration as a medical student, but doing this is no way obviates the necessity of passing five years in medical study after registration, so that, as a rule, people register as early as possible, hoping thereby to hasten the time when they shall become "qualified." In the case, however, of a precocious boy who is able to pass his Preliminary quite early—too early, in fact, for him to become a medical student at a great hospital, and who happens to be at school at some place where there are opportunities for studying these subjects—it would probably be desirable to keep him at school a year longer, and fill up his time clearing off the subjects of his First Examination. In any case, however, it is of the greatest importance that the student should pass this First Examination at the end of his first year of work, for nothing can alter the fact that he must spend four years in medical study after passing it before he qualifies. Any delay, then, in passing this First Examination means an extra year of study and an extra year of expense.

It is to be feared that in too many cases the new life, the new liberty, the escape from school, and the feeling that he is his own master lead the young student to neglect his earlier studies, and to waste time during his first year; but it cannot be too strongly impressed upon those on whom the expense of his medical education falls that any laxity which leads to failure at the First Examination at the appointed time means a proportionate lengthening of the course and increase of the expense. A kindly supervision over the young student during his first year, and even a certain amount of private "grinding"—anything that will make him take seriously to his work from the beginning—will pay in the end, for it must be confessed that the expense of a medical education is not merely what appears on the prospectus. To a large extent it arises from failing to pass the stated examinations at the stated times, and there can be no doubt that this also is the cause which leads to many of those sad cases in which medical students "drop out" and are lost sight of before they have reached a qualification. They fail to pass, and the money is not forthcoming to pay for the longer curriculum thereby entailed, and so all is lost. The money already spent, the time already given is all absolutely wasted, for unless the money and the effort devoted to the study of medicine end in a qualification they might as well have been thrown into the gutter.

Students in their earlier years do not seem to attach half enough importance to failure in examination. There is a theory abroad that the examiners are so hard that it is no disgrace to be "ploughed" now and again. But it vastly increases the expense of a medical education, and may lead to a complete throwing away of some of the best years of a man's life. If a student's means are limited so that he cannot extend his course, it would often be far better, if he finds the examinations more than he can do, that he should throw it all up and take to something else than that he should be a drag on his family for year after year, and end by being a "waster." After passing his First Examination the student goes on to the study of anatomy and physiology. In the

London schools it is usual to begin dissecting even before passing the First; but in any case a full year must be spent in this work, and then, if possible, the Second Examination must be passed. Then at the end of this year, that is two years after registration, the student, if successful, enters the wards, and having been grounded in a scientific knowledge of the human frame, he now at last commences his life work—the study of the diseases to which the human frame is liable. It is when this stage is arrived at that the real vital interest of medical study begins. There is still plenty of book work to be done, there are a perfect plethora of lectures to be attended, and the number of subjects to be taken up is embarrassing enough—so numerous and so embarrassing that the only possible chance of getting through them is by keeping strictly to the course of study laid down; but all these subjects have now to do with actual patients, they have a direct bearing on one's future work, and thus they become matter of absorbing interest. There is now a long interval before the Final Examination comes on. At last the hated and ever-present vision of the examiner can be put aside, at any rate for a time, and the student feels that he can give himself up untrammelled to his real work. There is, however, still a danger lest he should be tempted to specialise too early, and thus to neglect that even balance of knowledge which alone will take him safely to the goal he aims at, namely, qualification. Again then, even in his final stage, and with all his added wisdom, the student must not throw aside the teaching of experience, as expressed in the course of studies laid down in the regulations and advised by the authorities of his college. The Dean of the school may sometimes seem a rather autocratic person, but it is only by following the curriculum carefully and laboriously throughout that one can hope to get into the allotted time all the subjects that the examiners require. Erratic studies hardly count.

